# A diet rich in omega-3 fatty acid improves periodontitis and tissue destruction by MMP2- and MMP9-linked inflammation in a murine model

**DOI:** 10.1007/s10266-023-00831-y

**Published:** 2023-06-28

**Authors:** Patricia González-Alva, Diana Laura Solís-Suárez, Saúl Ernesto Cifuentes-Mendiola, Ana Lilia García-Hernández

**Affiliations:** 1 Laboratory of Tissue Bioengineering, Faculty of Dentistry, National Autonomous University of Mexico, Mexico City, Mexico; 2 Laboratory of Dental Research, Section of Osteoimmunology and Oral Immunology, FES Iztacala, National Autonomous University of Mexico, A. Jiménez Gallardo SN, San Sebastián Xhala, 54714 Cuautitlán Izcalli, Mexico; 3 Postgraduate Course in Dental Sciences, National Autonomous University of Mexico, Mexico City, Mexico

**Keywords:** Ligature-induced periodontitis, *Porphyromonas gingivalis-*infected ligature, Fish oil supplementation, Omega-3 fatty acids, Matrix metalloproteinases

## Abstract

Periodontitis is an oral-cavity inflammatory disease and is the principal cause associated with tooth loss. Matrix metalloproteinases 2 and 9 (MMP-2 and MMP-9) are important proteases involved in periodontal tissue destruction. The omega-3 polyunsaturated fatty acids (ω-3 PUFA) have been demonstrated to possess immunoregulatory properties in periodontitis. The aim of the study was to investigate the effects of ω-3 PUFA on inflammation and on the expression of MMP-2 and -9 in a murine periodontitis model. Twenty-four male C57BL/6 mice were divided into control mice (Control), control mice treated with ω-3 PUFA (O3), mice with periodontitis (P), and mice with periodontitis treated with ω-3 PUFA (P + O3). ω-3 PUFA were administered orally once a day for 70 days. Periodontitis in mice was induced by *Porphyromonas gingivalis-*infected ligature placement around the second maxillary molar. The mice were sacrificed, and blood and maxillary samples were collected. Flow cytometry was used to quantify tumor necrosis factor-alpha (TNFα), interleukin (IL)-2, IL-4, IL-5, and interferon-gamma. Histologic analysis and immunohistochemistry for MMP-2 and -9 were performed. The data were statistically evaluated using analysis of variance (ANOVA) and the Tukey post hoc test. Histological analysis showed that ω-3 PUFA supplementation prevented inflammation and tissue destruction and revealed that bone destruction was more extensive in the P group than in the P + O3 group (*p* < 0.05). Also, it decreased the serum expressions of TNFα and IL-2 and the tissue expression of MMP-2 and -9 in the periodontitis-induced model (*p* < 0.05). ω-3 PUFA supplementation prevented alveolar bone loss and periodontal destruction, probably by decreasing the expression of MMP-2 and MMP-9 and its immunoregulatory properties.

## Introduction

Periodontitis is a chronic inflammatory condition of the oral cavity that causes tissue destruction and eventually tooth loss. It is the sixth most prevalent disease worldwide and the first cause of tooth loss in adults [1]. Although the presence of gram-negative bacteria in the gingival sulcus is required in disease pathogenesis, all patients are not equally affected, and their response to treatment varies [2]. The host inflammatory response to the dysbiotic microbial community drives the immune cell-mediated hyper-inflammatory stage, resulting in tooth loss [2, 3]. Host modulation therapy has gained increasing interest in periodontal therapy. Hence, research on products containing docosahexaenoic acid (DHA) and eicosapentaenoic acid (EPA) is substantial. Long-term ingestion of Omega-3 polyunsaturated fatty acids (ω-3 PUFA) is associated with a better periodontal condition. In contrast, a decreased intake of ω-3 PUFA is associated with greater severity of periodontal disease [3]. The intake of ω-3 PUFA can reduce the secretion of proinflammatory cytokines, promote lipogenesis, and reduce serum triglyceride levels, all of which are related to the development of cardiovascular and inflammatory diseases, including periodontitis [2, 4–6].

It has been suggested that a reduction in gingival and periodontal inflammation can be achieved with an optimized diet alone, without a change in oral-hygiene habits [7]. While periodontitis treatment should include an interdisciplinary effort, therapeutic approaches focusing solely on plaque reduction may be insufficient due to the persistence of chronic inflammation [3]. Hence, periodontitis treatment should increasingly focus on modulation of the immune response [2]. As for ω-3 PUFA, previous reports indicate that these can inhibit the synthesis of lipid mediators of inflammation, modify the cellular functions of polymorphonuclear leukocytes, and modulate lymphocyte proliferation and cytokine production. Therefore, ω-3 PUFA have been proposed as adjuvant therapy for inflammation-bone loss prevention, including periodontitis [8]. In this regard, in a clinical study, patients treated for three months with EPA and DHA along with scaling root planning showed improved clinical attachment levels [9]. Previously reported studies conducted in rat models demonstrated the effectiveness of diet supplementation with ω-3 PUFA in modulating alveolar bone resorption following *Porphyromonas gingivalis (P. gingivalis)* infection, reducing inflammation, and promoting bone regeneration in apical periodontitis [6, 7].

The mechanism of action by which ω-3 PUFA improves alveolar bone loss is related to the decreased expression of pro-inflammatory cytokines, such as tumor necrosis factor alpha (TNFα), Interleukin (IL)-1β, and IL-6 in rats [10], probably via inhibition of lipoxygenase and cyclooxygenase due to the decrease in prostaglandin D2, prostaglandin E2, and leukotriene B4 in plasma after 4 weeks of ω-3 PUFA treatment [11]. Also, the inhibition of these two pathways resulted in decreased inflammation [6]. High levels of inflammatory mediators and cytokines in the systemic circulation accelerate the pathogenesis of bone resorption. DHA decreases bone resorption by suppressing the expression of NFATc1, TRAP, and osteoclastogenesis-related c-FOS genes [12]; downregulating RANK and vitronectin receptor expression; and inhibiting osteoclast migration [13].

Also, ω-3 PUFA treatment in rats for 6 months promotes mitochondrial maintenance of turnover through biogenesis or an autophagy mechanism as an antioxidant mechanism [14].

In periodontitis, activated monocytes and lymphocytes release matrix metalloproteinases (MMP), particularly MMP-9, an enzyme responsible for bone formation [1]. MMP-9 is secreted as a proenzyme; it remains inactive until activation by removal of the propeptide moiety by proteolytic enzymes, such as MMP-2 and stromelysin-1, among other MMPs. Also, MMP-9 is generally secreted in conjunction with a specific inhibitor, TIMP-1, which controls its proteolytic activity. The imbalance between MMP and their inhibitors leads to excessive degradation, expected in chronic inflammatory diseases, including periodontitis [15].

Also, a clinical placebo-controlled trial in individuals with generalized severe chronic periodontitis demonstrated a decrease in mean probing depth and clinical attachment gain after a supplementary diet with ω-3 PUFA and low-dose aspirin for 6 months. In addition, salivary levels of both RANKL and MMP-8 decreased [16]. The ligature infected with *P. gingivalis* comprises a protocol in which dynamic gingival tissue inflammation can be observed [17].

This study aimed to investigate the periodontal and immunological effects of daily supplementation with fish oil at 352.7 mg/kg body weight (BW) with ω-3 PUFA for 2.3 months as a supplementary diet, followed by the induction of periodontitis, because it has been pointed out that prolonged and prophylactic supplementation of ω-3 PUFA had a greater effect on bone metabolism [18, 19]. We hypothesize that the supplementation with ω-3 PUFA, in ligature-induced periodontitis, could reduce periodontal destruction by decreasing inflammatory markers and with MMP-2 and MMP-9 activation as a mechanism of action.

## Materials and methods

### Animals

Male C57BL/6 mice 6 weeks of age (*n* = 24) were obtained from the Faculty of Higher Studies Iztacala Vivarium. The mice were randomly divided into four groups: Control (*n* = 6); ω-3-PUFA-supplemented diet (O3; *n* = 6), Periodontitis (P; *n* = 6), and Periodontitis with a ω-3 PUFA-supplemented diet (P + O3; *n* = 6). The sample size and power was according to the calculation reported previously [20], considering previous reports of ω-3 in periodontitis mouse models [21, 22]. Mice were housed in polycarbonate boxes in a temperature-controlled environment with a 12-h light/12-h dark cycle and had free access to water and food (Laboratory Autoclavable Rodent Diet 5001). All procedures were performed under the recommendations of the Official Mexican Standard NOM-062_ZOO-1999 and were approved by the Ethics Committee of the FESI, UNAM (CE/FESI/062015/1047). We euthanized mice with intraperitoneal sodium pentobarbital (Aranda, Mexico; #0121) overdose. Maxillae and blood samples were collected and stored at − 80 $^\circ{\rm C}$ until use.

### Dietary supplement

**Fig. 1:**
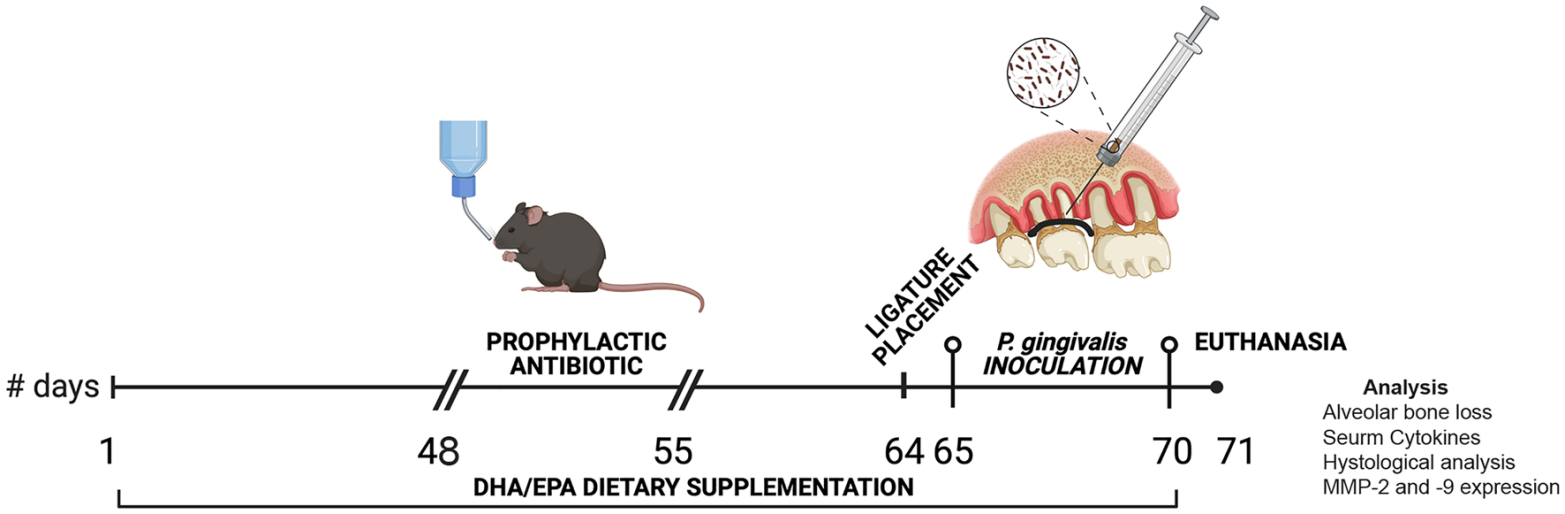
Timeline of the experiment

From day 1 until 12 h prior to sacrifice, mice were given daily intragastric (i.g.) gavage of 40 mg/kg of EPA/DHA (60% EPA, 40% DHA; GNC Fish Oil) for 70 days [6]. Each mouse was weighed before administration, and EPA/DHA doses were adjusted (Fig. 1).

### Bacterial strain

The *P. gingivalis* strain (ATCC #53,977) was used to induce periodontal infection. The bacteria were cultured on blood agar plates and maintained according to the manufacturer’s specifications. Strain purity was verified through colony characterization and gram-negative staining. Mice were infected with *P. gingivalis* orally (10^9^ live bacterial cells suspended in 2% carboxymethylcellulose in 100 µL of a phosphate-buffered vehicle) [23].

### Periodontitis induction

Forty-eight days after the start of dietary EPA/DHA supplementation, mice received prophylactic antibiotic treatment with 15 mL of Sulfamethoxazole/Trimethoprim; 200 mg/40 mg/5 mL diluted in 300 mL of water for 12 days in their drinking water to suppress the oral microbiota. Antibiotic treatment was suspended 4 days before inoculations. On day 64, P and P + O3 groups were anesthetized with a ketamine (10 mg/mL) and xylazine (1 mg/mL) solution (0.1 mL/10 g BW). Periodontitis induction lasted for a total of 7 days. Ligature placement was carried out according to a previously reported protocol [24]. Briefly, a 6–0 silk suture (Atramat^®^) was attached around the second maxillary molar and was tied gently to avoid damage to the periodontal tissue [24]. Then, oral inoculations with *P. gingivalis* were performed on the mucogingival junction adjacent to each ligature. The inoculations were administered for 7 days in the following order: at 24 h and at 72 h after ligature placement, followed by three consecutive inoculations every 24 h. Mice were held without food or water for 1 h after inoculations to minimize elimination from the oral cavity, based on the protocol reported by Bendyk et al. [22]. On day 71, all groups were sacrificed (Fig. 1).

### Alveolar bone loss assessment

Bone loss was measured according to Abe and Hajishengallis [24]. Briefly, the right hemimaxillae were boiled for 10 min, cleaned of soft tissues, and then bleached for 5 min. The hemimaxillae were stained with 0.5% eosin and 1% methylene blue. Periodontal bone loss was measured by positioning an endodontic explorer on the cusps and grooves of each molar on the palatal and buccal surfaces of the maxillae. Periodontal bone heights were measured as the distances from the cementoenamel junction (CEJ) to the alveolar bone crest (ABC) in different anatomical referents of the dental crown of the buccal and palatal surfaces of the three maxillary molars: on the mesio-palatal cusp, palatal groove, disto-palatal cusp, disto-palatal groove, mesio-buccal cusp, buccal groove, disto-buccal cusp, and disto-buccal groove of the first molar; on the mesio-palatal cusp, palatal groove, disto-palatal cusp, mesio-buccal cusp, buccal groove, and disto-buccal cusp of the second molar; and on the palatal and buccal cusp of the third molar. The total CEJ − ABC distance for the groups with periodontitis was calculated and subtracted from the CEJ − ABC distance obtained in the control group. The distances were transported to an analog vernier caliper (14,394, TRUPER^®^), with 0.02-mm precision and expressed in mm. We calculated the area under the curve to obtain the area of global bone loss.

### Cytokine detection

The serum concentrations of IL-2, IL-4, IL-5, IFNγ, and TNFα were measured at the end of the experiment (71 days) with a BD Cytometric Bead Array (CBA) Mouse Th1/Th2 Cytokine Kit (BD Biosciences, #551,287) according to the manufacturer’s instructions in a FACS Aria III (BD) flow cytometer.

### Histological analysis

Left hemimaxillae were fixed in 4% paraformaldehyde for 48 h and then decalcified in an EDTA 7% solution. The hemimaxillae were embedded in paraffin. Serial Sects. (4 µm in thickness) were then prepared and routinely stained with Hematoxylin and Eosin (H&E) to determine the inflammation grade; Sirius Red/Fast Green double stain was utilized for periodontal collagen-fiber assessment according to their thickness, and immunochemistry was employed to detect the expression of MMP-2 and -9. The slides were analyzed under an optical microscope (AmScope).

### Inflammation grade

Inflammation infiltrate was evaluated for its intensity and extension. The average number of cells per field was noted, as well as the extension of inflammation into the periodontal ligament area adjacent to the ligature site. The inflammatory cells with a polynuclear structure and a morphological characteristic of neutrophils, macrophages, and plasma-like cells were quantified. For each experimental condition, a total area of A = 2.56 cm^2^ was examined, and the cell count per unit area was calculated. The number of cells per field was calculated as the average of four separate fields (40 ×) magnification (*n* = 20 fields) for each experimental condition. The intensity of the inflammatory infiltrate was graded as follows: absent (0 to a few inflammatory cells); mild (< 25 inflammatory cells); moderate (25–125 inflammatory cells), and severe (< 125 inflammatory cells).

### Collagen fiber assessment

For the Sirius Red/Fast Green double stain, histological sections were incubated in 0.04% Fast Green (Sigma-Aldrich, #104,022) for 15 min, washed with distilled water, and then incubated in 0.1% Fast Green and 0.04% Sirius Red (Direct red 80, Sigma-Aldrich, #365,548) in saturated picric acid for 30 min. Periodontal collagen fibers adjacent to the maxillary second molars were assessed according to their thickness. On examination, Sirius Red staining, which stains collagen types I, II, and III, detected small amounts of collagen with high sensitivity. The larger collagen fibers appeared bright red or orange, and the thinner ones appeared green. Also, collagen fibers appeared red/pink in color and were classified as dense, thin, or mixed (predominantly dense or predominantly thin) [25].

### MMP-2 and MMP-9 detection

The slides were deparaffinized, rehydrated, and washed with ImmunoDNA washer 1X (Bio SB, Inc.). For antigen retrieval, slides were boiled in a citrate buffer (0.06 M; pH 6) for 15 min and then blocked with a peroxidase solution (0.3% H_2_O_2_/Methanol) for 20 min. The slides were incubated with BSA-PBS (2% Tween-20 [11332465001]/Bovine Serum Albumin [A7030], Sigma-Aldrich^®^) for 30 min, and primary antibodies (1:100 dilution) for MMP-2 (SC-10736; Santa Cruz Biotechnology) and MMP-9 (SC-10737; Santa Cruz Biotechnology) at 4 $^\circ{\rm C}$ overnight. The reaction was revealed with the Mouse/Rabbit ImmunoDetector Biotin HRP Label Kit (Bio SB, Inc.) according to the manufacturer’s specifications. Mayer’s hematoxylin (C.I. 752, SIGMA-ALDRICH^®^) was used for counterstaining.

Immunohistochemical evaluation was conducted in four fields, taken from experimental groups samples at 40 × magnification with an optical microscope (AmScope). A total of 20 fields were analyzed for each experimental group (*n* = 20 fields per group). The immunohistochemical evaluation was assessed with Fiji ImageJ software and with the aid of the IHC toolbox and the ITCN plugin and presented as a percentage of the positive area and pixel intensity. Briefly, the image was acquired with the AmScopeX software. Then, the optimal threshold for positive pixels that corresponded to the areas of DAB immunohistochemistry was determined using Fiji ImageJ software by changing the hue value and color saturation employing the original image for comparison. Subsequently, a binary image was produced, and the positive area was expressed as a percentage (positive pixels to total pixel area). Finally, the same fields were used to quantify the intensity of the staining. The intensity was quantified with the aid of the IHC plugin in positive cells and presented as pixel intensity minus the intensity of the background. Microphotographs were taken with an Axiocam microscope camera (AmScope Microscopy, LLC, USA).

### Statistical analysis

The data from each analysis was evaluated by the D’Agostino-Pearson test for normality. Two-way analysis of variance (ANOVA) with Tukey's post hoc multiple comparison test was performed for alveolar bone loss and serum cytokines. A descriptive histological analysis was performed to evaluate the inflammation grade and collagen fibers in the periodontal tissue. For immunohistochemistry, in which an area of 2.0165 × 104 µm^2^ was evaluated for each field (20 fields of view per mouse) one-way analysis of variance (ANOVA) with Tukey’s post hoc multiple comparison test was performed. We used the software GraphPad Prism (version 9.0). Data are presented as the mean ± standard error of the mean (SEM). Significant differences are listed in each analysis. Differences were considered statistically significant if *p* ≤ 0.05.

## Results

### A diet supplemented with omega-3 PUFA decreases bone loss in ligature-induced periodontitis

**Fig. 2:**
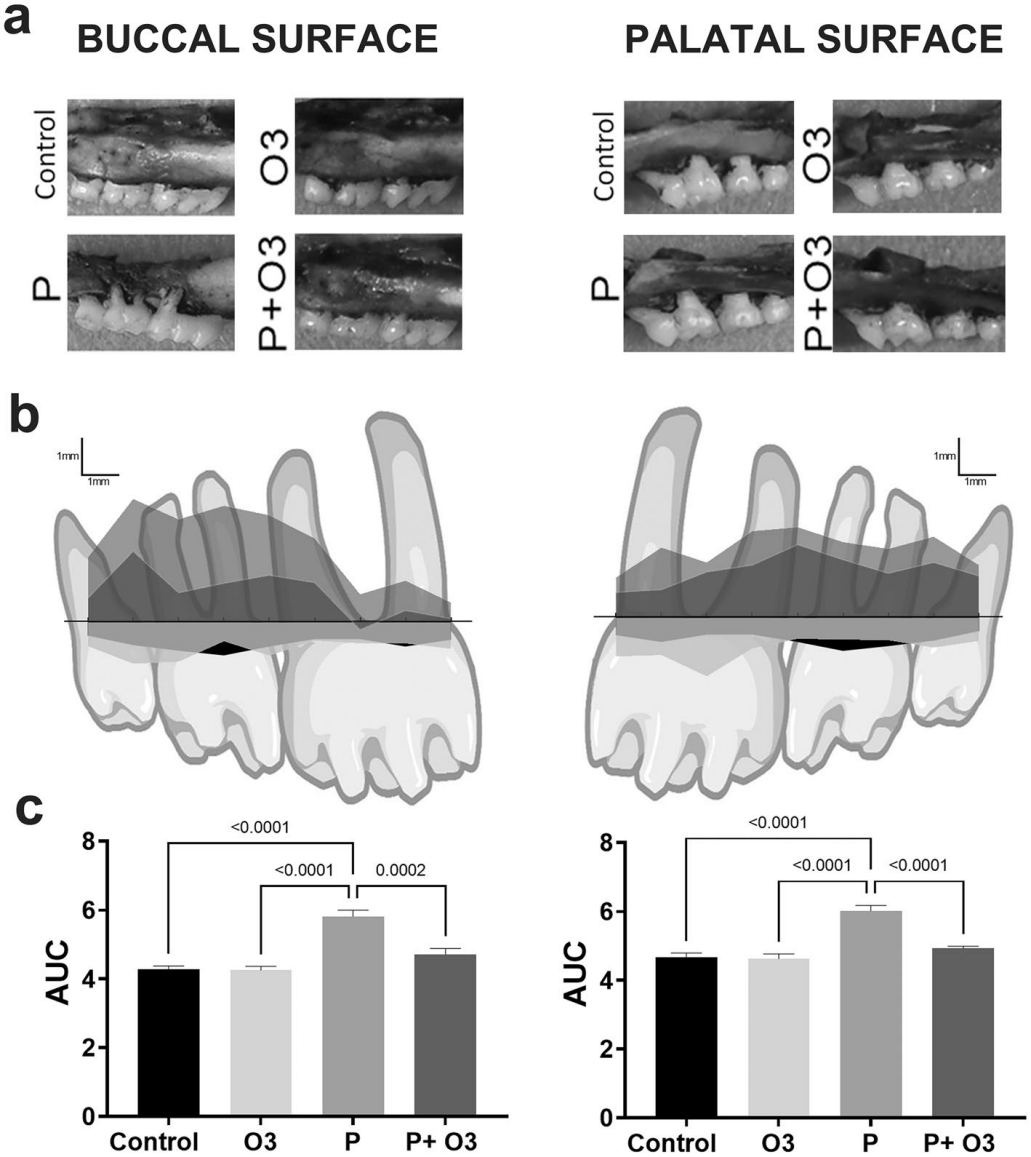
Alveolar bone loss. **a** Representative images of the buccal and palatal hemimaxillae of each study group. **b** Graphic representation of the mean 8-site total CEJ-ABC distance measurements at the buccal and palatal of the four groups. **c** Area Under the Curve (mean and standard error of the mean) of the histogram obtained in 8-site total CEJ-ABC distance measurements at the buccal and palatal of the four groups. The bracket indicates the* p* value for the difference between experimental groups, ANOVA F (16, 135) = 2.682

Alveolar bone loss in mice with ligature-induced periodontitis caused by *P. gingivalis* was evaluated by comparing mice supplemented with ω-3 PUFA and control mice without supplementation. As observed in Fig. 2, a lower bone crest was found on the P group’s buccal and palatal surfaces compared with the Control (*p* < 0.0001) and O3 (*p* < 0.0001). The P + O3 group showed that the area of bone loss was reduced compared to that of the P group on the buccal surface (*p* = 0.0002) and the palatal surface (*p* < 0.0001) (ANOVA F (16, 135) = 2.682, *p* = 0.0010). No statistical or clinical differences were observed between the Control and the O3 group.

### A diet supplemented with omega-3 PUFA decreases serum levels of inflammatory cytokines

**Fig. 3:**
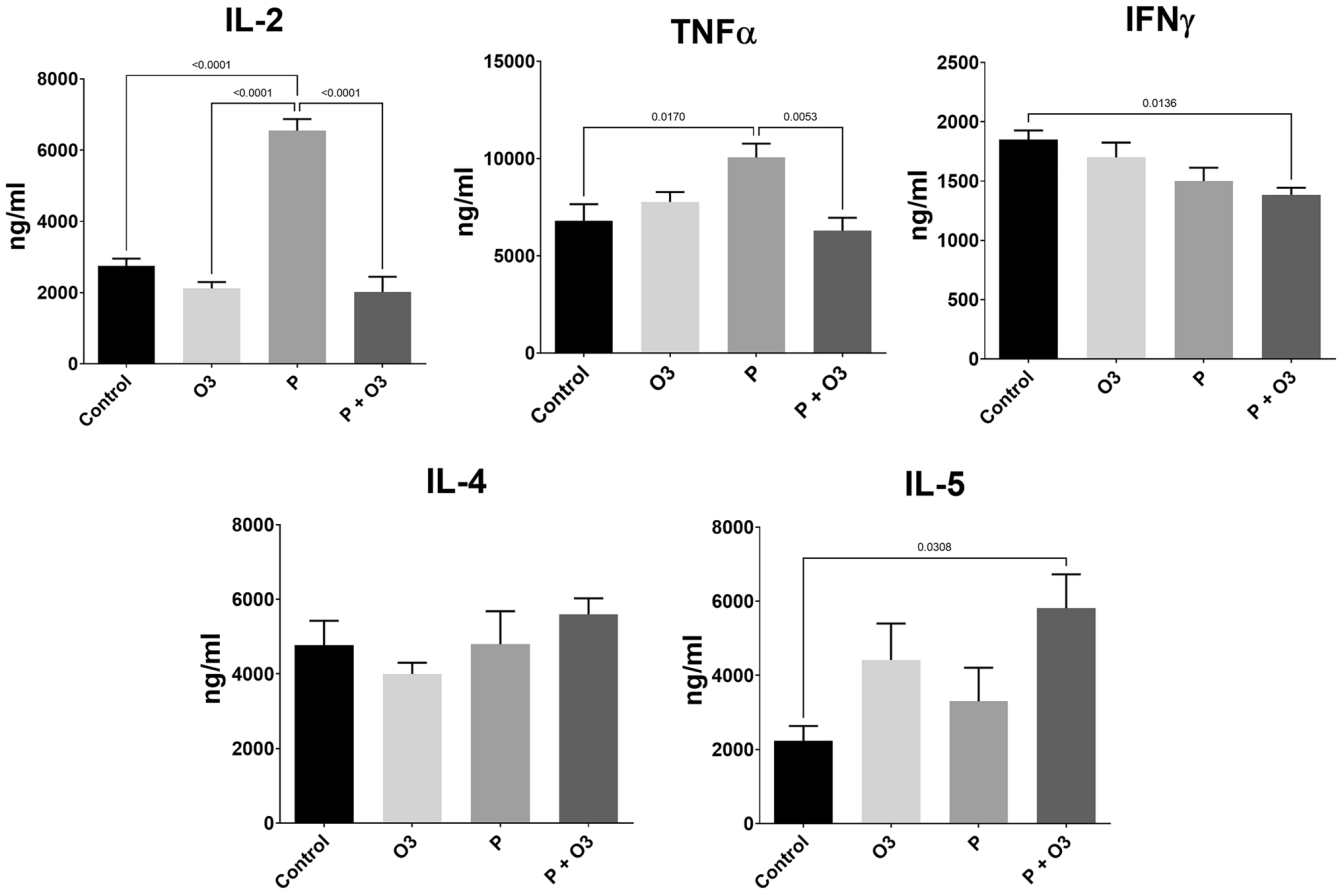
Serum cytokine levels of TNFα, IL-2, IFNγ, IL-4, and IL-5. The bracket indicates the *p* value; the data are presented as the mean and standard error of the mean

We evaluated the effects of ω-3 PUFA on the cytokines TNFα, IL-2, IFNγ, IL-4, and IL-5 in a *P. gingivalis* ligature-induced periodontitis in the serum of mice. The results are presented in Fig. 3. The levels of TNFα and IL-2 were increased in the P group compared to the Control (TNFα, *p* = 0.0170; IL-2, *p* < 0.0001) and the P + O3 (TNFα, *p* = 0.0053; IL-2, *p* < 0.0001) group. In this regard, ω-3 PUFA supplementation decreased the serum levels of IL-2 and TNFα in the P + O3 group compared to the P group (TNFα, *p* = 0.0053; IL-2, *p* < 0.0001). Additionally, we observed an increase in IL-5 (*p* = 0.0308) and a decrease in INFγ ( *p* = 0.0136) in P + O3 mice compared to the control group (IL-2, ANOVA F (3, 20) = 50.86, *p* < 0.0001; TNFα, ANOVA F (3, 20) = 5.758, *p* = 0.0052; INFγ ANOVA F (3, 20) = 4.599, *p* = 0.0132; IL-5 ANOVA F (3, 20) = 3.374, *p* = 0.0387). No statistical differences were found in IFNγ, IL-4, and IL-5 between the P and the P + O3 groups.

### A diet supplemented with omega-3 PUFAs prevented bone destruction and inflammation induced by experimental periodontitis

**Fig. 4:**
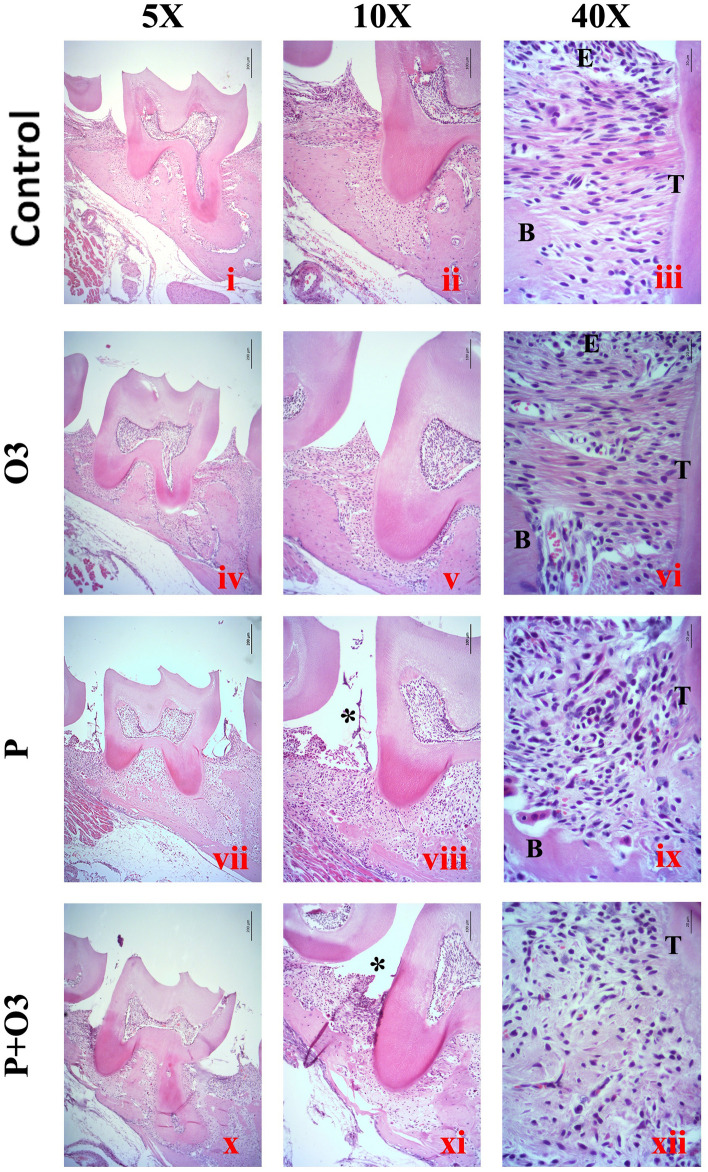
Representative images of Hematoxylin and Eosin (H&E) staining. (Fig. 4i–iii): Samples from the Control group (Control) show epithelial cells arranged in close contact and organized into stratified layers. The interdental gingiva between the first and second molars is well defined, composed of squamous epithelium, and supported by an intact basement membrane; the underlying connective tissue is composed of parallel collagen fibers, small fibroblasts, and vessels of small diameter; a thick ridge of bone tissue, representative of the alveolar bone, can be observed. (Fig. 4iv, v): The microphotographs of the Omega-3 group (O3) revealed similar characteristics to the Control group; at the epithelial-connective tissue interface, cells are cuboidal in morphology, with their nuclei located near the basal membrane and facing the underlying connective tissue, composed of dense, well-organized collagen fibers, small fibroblasts, and blood vessels of small diameter. (Fig. 4vii–ix): Tissue samples from the periodontitis (P) group and adjacent to the localization of the ligature (*) exhibited a stratified epithelium and shedding of the superficial layers. (Fig. 4viii): Edematous changes can be observed in all epithelial layers. The underlying connective tissue in the P group demonstrated edema and enlarged blood vessels filled with erythrocytes and inflammatory cells. The alveolar bone is observed as a thin ridge. (Fig. 4ix): Adjacent to the alveolar-bone lining, there are large, osteoclast-like multinucleated cells. (Fig. 4x, xi): The periodontitis + Omega3 (P + O3) group revealed inflammation near the ligature area (*); however, the alveolar bone is thicker than observed in the P group. The inflammatory infiltrates in the P, and P + O3 groups were composed of a mixed population of polymorphonuclear leukocytes, plasma cells, and macrophages. Interestingly, plasma cells were dominant in many cases, while collagen fibers and fibroblast were scarce. (B) Bone, (T) Tooth, (E) Epithelium. Scale bar 5 × (200 µm), 10 × (100 µm), and 40 × (20 µm)

Histological analysis was performed to confirm the development of periodontitis in a *P. gingivalis*-infected ligature model. Analysis of the region between the first and second molars of the Control mouse group demonstrated tissue presenting with a normal periodontium where the gingiva, periodontal ligament, alveolar bone, and cementum are defined (Fig. 4i–iii). Tissue analysis revealed mild to severe inflammatory-cell infiltrates coupled with areas of bone destruction, edematous changes, and blood-vessel congestion in the P and P + O3 groups (Fig. 4vii–ix; Table 1). Supplementation with ω-3 PUFA prevented bone destruction and inflammation induced by experimental periodontitis (Fig. 4x–xii), with the mice revealing absent to mild inflammation (Table 1). Interestingly, in one sample of the P + O3 group, inflammation was absent. Furthermore, the histopathological analysis demonstrated that inflammatory infiltration and bone destruction were more severe in the P group than in the P + O3 group (Table 1).

**Table 1:** Scores of Inflammatory Cells according to the Groups

Intensity of inflammatory infiltrate	Groups			
	control	Periodontitis	P + Omega3	Omega3
1. Absent inflammation	3/5		1/5	2/5
2. Mild inflammation	2/5	1/5	3/5	3/5
3. Moderate inflammation		3/5	1/5	
4. Severe inflammation		1/5		
Total number of cells/fields*	213	198	201	205

Area 2.0165 × 104 µm^2^/field

*P* + *Omega3* periodontitis with ω-3 supplementation

*Mean of four fields per sample, *n* = 20 fields per group

### A diet supplemented with omega-3 PUFA preserves the collagen fibers of the periodontal tissues

**Fig. 5:**
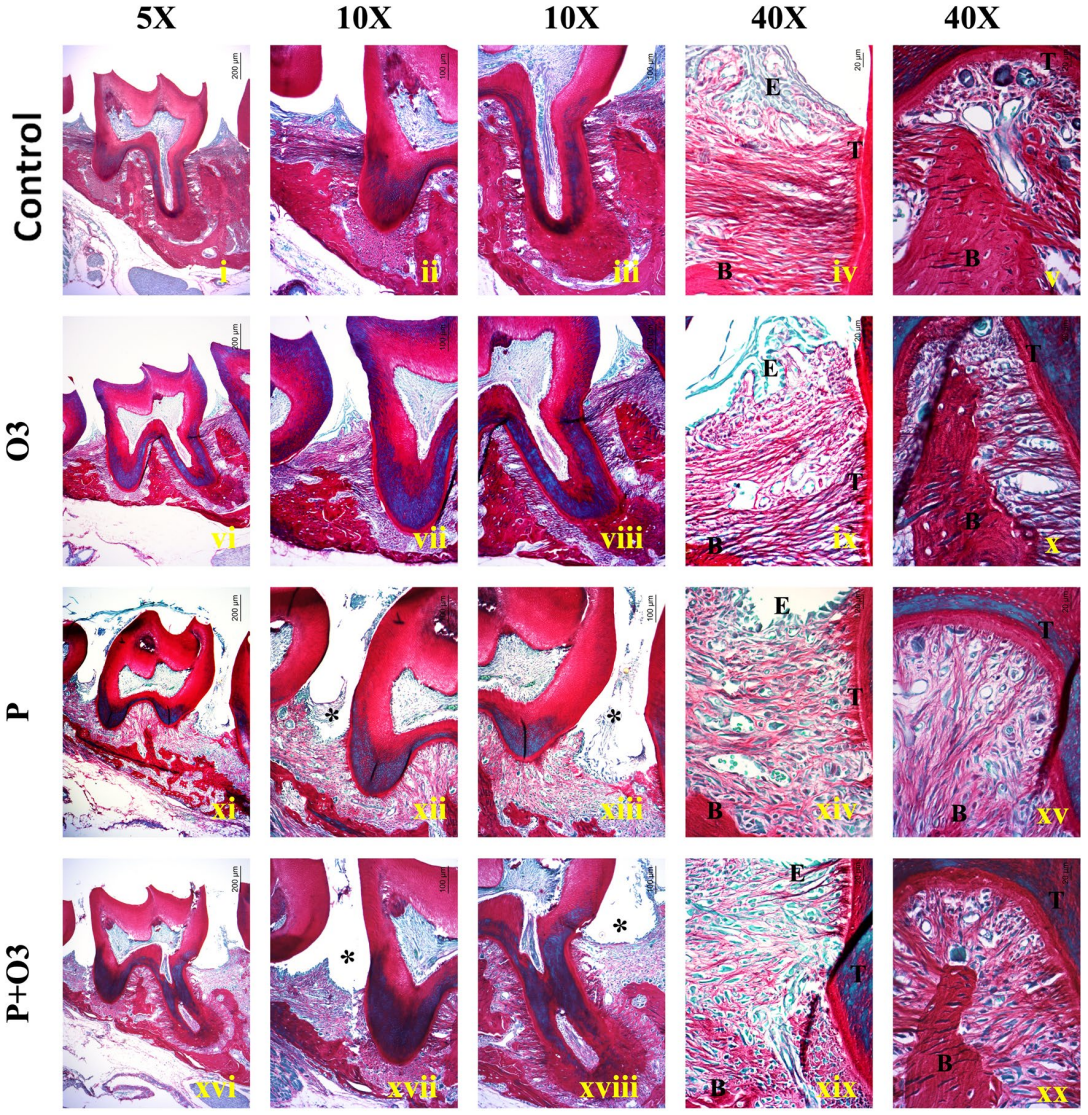
The Control group shows predominantly dense and well-organized fibers stained bright red; in Fig. 5iv, the fibers run parallel to the basal membrane of the oral Epithelium (E), with densely spaced fibroblasts, and in Fig. 5v, vessels of medium and small diameter can be observed. Furthermore, the fibers bind to the alveolar bone and to the Tooth (T) cementum; (Fig. 5vi–x): the Omega-3 (O3) group show a similar staining pattern, in which the parallel collagen fibers are bright red, and fibroblasts cells are also densely spaced (Fig. 5xi–xv): the group of induced periodontitis (P) reveal thin, disorganized fibers stained light green under the area of the ligature (*), with prominent bone resorption demonstrated by small and thin fragments of mineralized tissue under the apex of the tooth roots (Fig. 5xi and xii). In Fig. 4xiv, a shedding and thin epithelium can be observed in the P group. Also, vessels of a large, medium, and small diameter filled with blood cells are present in the P group. Figure 5xvi–xx, while the Periodontitis + Omega-3 (P + O3) group showed a mixed pattern of staining with red and light-green fibers; in Fig. 5xvii and xviii, the bone ridge remains visible and supports more than two-thirds of the root. Scale bar 5 × (200 µm), 10 × (100 µm), and 40 × (20 µm)

Samples of the P group exhibited a predominance of a light-green color in the tissue underlying the oral epithelium and around the periodontal ligament, arranged in a disorganized manner and of thin morphology (Fig. 5xi–xv). By contrast, the Control and O3 groups showed a predominant red–orange color, characteristic of dense fibers organized in a parallel pattern, and a regular and well-distributed morphology under the oral epithelium and adjacent to the tooth cementum (Fig. 5i–x). As for the P + O3, it is shown that the areas near the alveolar bone exhibited predominantly dense red fibers; however, thin light-green fibers can be observed near the ligature region (Fig. 5xvi–xx).

### A supplementary diet with omega-3 PUFA decreases the expression of MMP-2 and -9

**Fig. 6:**
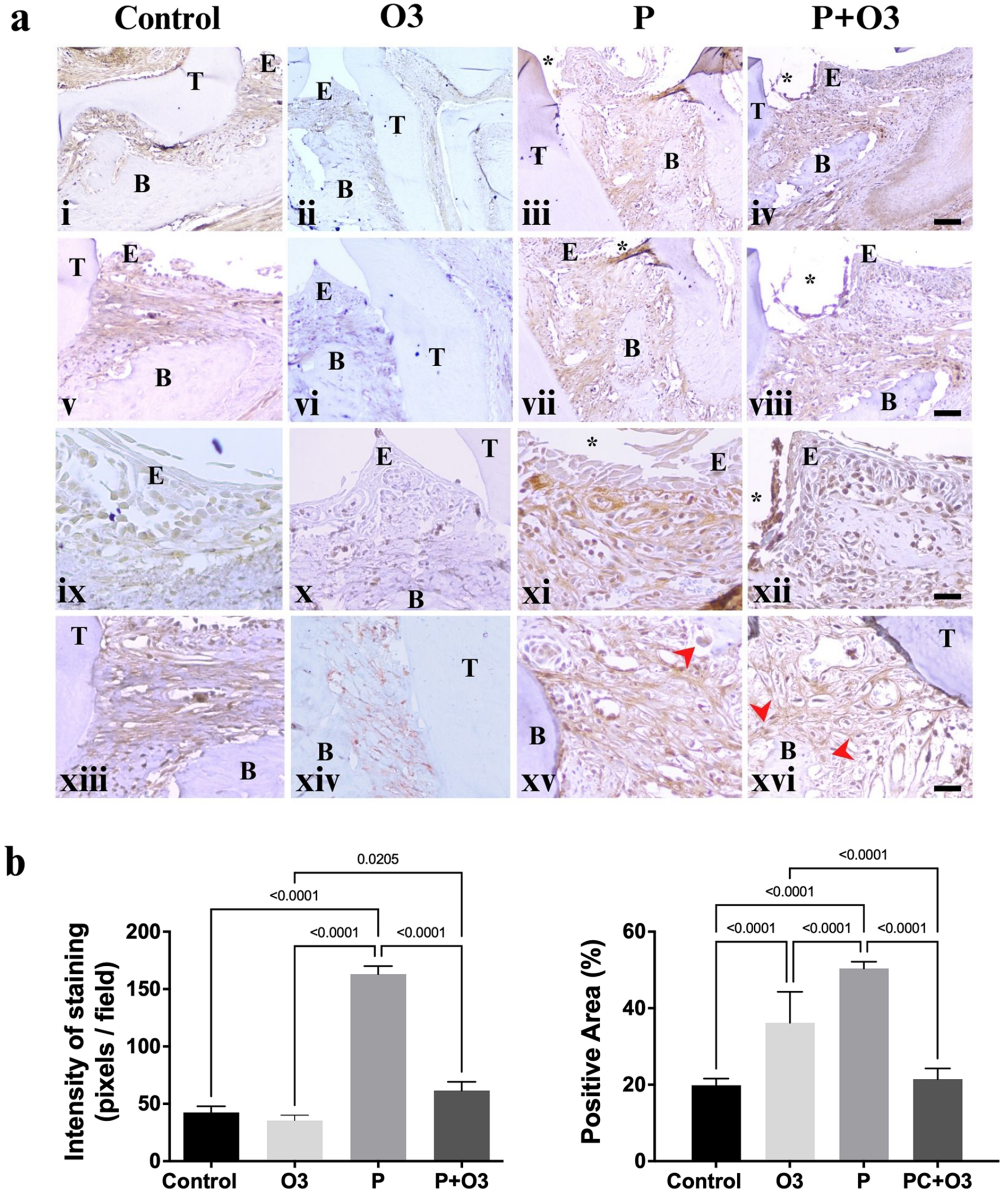
MMP-2 immunohistochemistry of murine periodontal tissues. **a** Representative images of the MMP-2 staining control group (Control), Fig. 6i, v, ix, and xii showed that periodontal tissues are slightly positive for MMP-2 staining in polymorphonuclear cells, plasma cells, and some macrophages. The pattern of expression was cytoplasmatic with rare nuclear staining in positive cells. A similar expression pattern was observed in the group with Omega-3 supplementation (O3). Stronger staining was observed in the Periodontitis group (P), with a more robust reaction near the *P. gingivitis*-infected ligature site, Fig. 6iii, vii, xi, and xv. Mild staining was observed in the epithelium and in the inflammatory cells of the periodontitis-induced group with Omega-3 supplementation (P + O3), Fig. 6iv, viii, xii, and xvi. Occasionally, enlarged fibroblast-, endothelial-, and osteoclast-like cells were also positive for the MMP-2 antibody (red arrows) near the areas with bone (B) destruction. A higher staining density was observed in the P + O3 group; however, there was no difference in expression between the Omega-3 and Control groups. **b** Graphic representation of the staining intensity and the percentage of the positive area of all examined groups stained with the MMP-2 antibody. A: (*) ligature region, (T) tooth, (B) bone, (E) epithelium. (Fig. 6i–iv) magnification 10 × , scale bar 200 µm; (Fig. 6v–viii) magnification 20 × , scale bar 100 µm; (Fig. 6ix–xvi) magnification 40 × , scale bar 20 µm. B: The bracket indicates the* p* value of the ordinary-one-way ANOVA test, the graphs represent the mean and standard error of the mean

The periodontal tissues of mice presenting with experimental periodontitis receiving no treatment demonstrate marked staining for MPP-2 and -9 markers compared to the periodontal tissues of the Control, Omega 3, and the treated group (P + O3) (Figs. 6i–xiii and 7i–xiii).

**Fig. 7:**
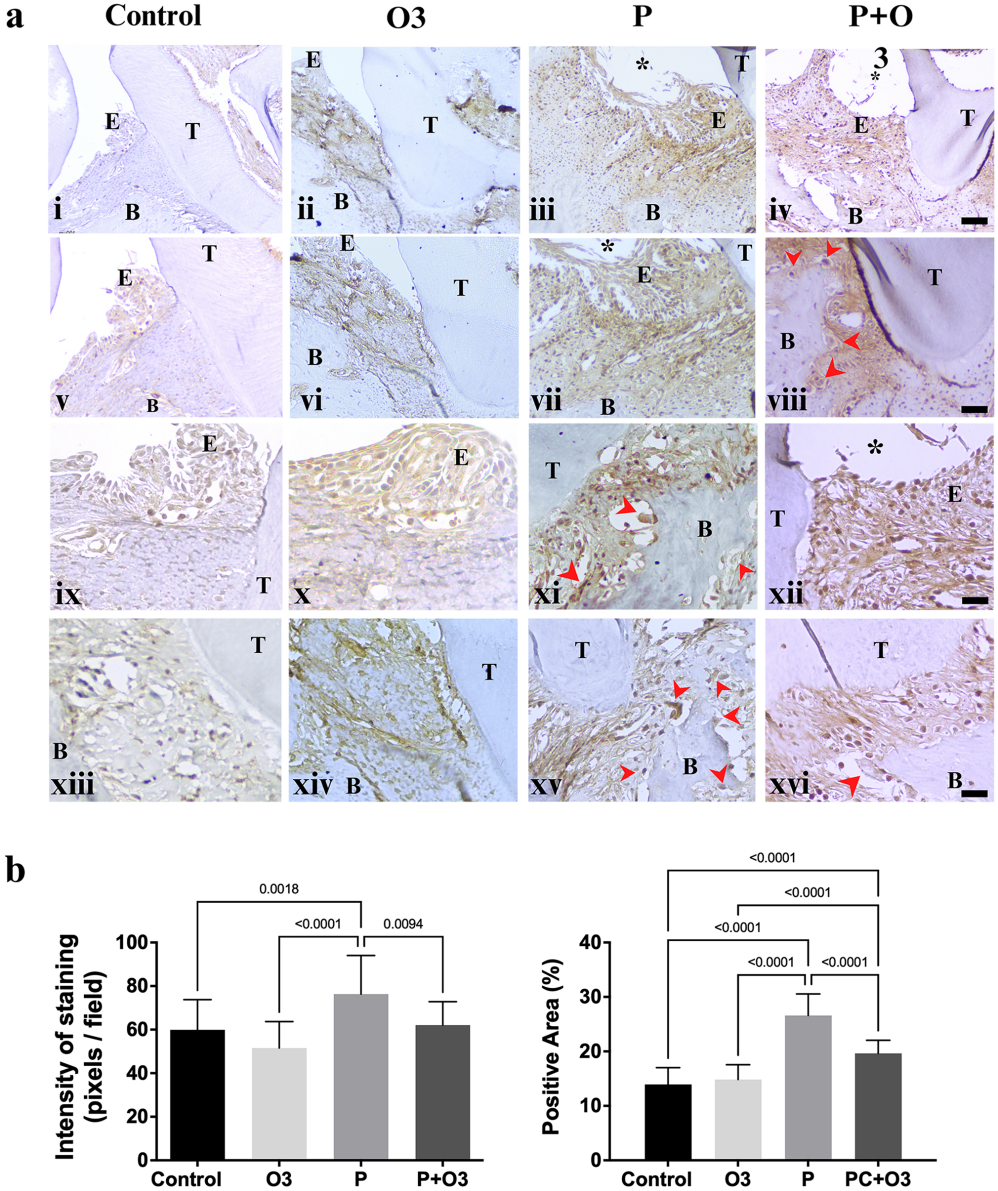
MMP-9 immunohistochemistry of murine periodontal tissues. **a** The representative images of the slides stained with the MMP-9 antibody. Microphotographs show that positive cells were mainly detected in periodontal ligament infiltrated with inflammatory cells near the oral epithelium (E), enlarged fibroblasts in the underlying connective tissue of the ligature area, and osteoclast-like cells (red arrows) near the bone (B). Osteoclast-like cells near the bone destruction showed stronger positive staining in the Periodontitis group (P) than in the Periodontitis group treated with Omega-3 (P + O3). Stronger staining was observed in the P group (Fig. 7iii, vii, xi, and xv); by contrast, mild or moderate staining was observed in the Periodontitis group treated with Omega-3 (P + O3) (Fig. 7iv, viii, xii and xvi), the Control (Fig. 7i, v, ix, xiii), and the Omega-3- supplemented diet group (Fig. 7ii, vi, x, and ixv). **b** Graphic representation of the staining intensity and the percentage of the total area that was positive in all the examined groups stained with the MMP-9 antibody. **a** (*) Ligature region, (T) tooth, (B) bone, (E) epithelium. (i–iv) magnification 10 × , scale bar 200 µm; (Fig. 7v–viii), magnification 20 × , scale bar 100 µm; (Fig. 7ix–xvi) magnification 40 × , scale bar 20 µm. **b** Control compared with the periodontitis-induced group; periodontitis-induced group compared with the treatment group, and the Omega-3-supplemented group. The bracket indicates the *p* value of the ordinary-one-way ANOVA test, the graphs represent the mean and standard error of the mean of 20 fields per group at 400 × magnification

Furthermore, the immunohistochemistry of murine periodontal tissues revealed that MMP-2-positive cells were mainly detected in periodontal ligaments infiltrated with inflammatory cells and in bone-destruction areas. With respect to expression, cytoplasmic and, rarely, nuclear staining were observed in polymorphonuclear cells and plasma-like cells. Analysis of the staining intensity revealed a high expression in the P group (*p* < 0.0001) versus the Control group (Fig. 6a, b). MMP2 expression was decreased in the P + O3 versus the P group (*p* < 0.0001) (ANOVA F (3, 76) = 90.70, *p* < 0.0001) (Fig. 6a, b). The percentage of the positive area of MMP-2 was higher in the P group compared with the Control (*p* < 0.0001) and P + O3 (*p* < 0.0001) groups (ANOVA F (3, 76) = 205.9, *p* < 0.0001) (Fig. 6a and b).

Regarding the MMP-9 immunohistochemistry, the representative images of the stained slides with the MMP-9 antibody are presented in Fig. 7. Microphotographs revealed that positive cells were mainly detected in periodontal ligaments infiltrated by inflammatory cells and in bone-destruction areas. The staining pattern in positive cells was cytoplasmic and rarely nuclear and was found in polymorphonuclear cells, plasma-like cells, and osteoclast-like cells near bone-destruction areas.

The intensity of expression also varied between groups, with higher expression detected in the P group versus the Control group (*p* = 0.0018), MMP9 expression was decreased in the P + O3 versus the P group (*p* < 0.0094) (ANOVA F (3, 76) = 11.05, *p* < 0.0001) (Fig. 7a, b). The percentage of the total area that was positive in the MMP-9 group was higher in the P versus the control group (*p* < 0.0001); versus the P + O3 (*p* < 0.0001); and versus the O3 (*p* < 0.0001) group (ANOVA F (3, 76) = 70.36, *p* < 0.0001) (Fig. 7a, b).

## Discussion

Periodontitis is a chronic disease characterized by the progressive loss of the attachment of gingival tissue, and it destroys the periodontal ligament and adjacent supporting alveolar bone [26, 27]. With regard to the use of ω-3 PUFA in dentistry, the majority of publications in the literature investigate individuals with periodontitis. A recent review by Azzi et al. estimated the effects of ω-3 PUFA and a ω-3-rich supplementary diet on the severity of periodontitis [28]. These authors concluded that increased EPA and DHA levels in the plasma could lead to a decrease in the progression of periodontitis [3, 28].

The heterogeneity of clinical data renders it challenging to allow conclusions regarding the adjunctive use of ω-3 on periodontal health. The present work evaluated the supplementary diet with ω-3 PUFA in a murine periodontitis model, and with the approach in our protocol, dynamic inflammation − destruction of gingival tissue can be observed. Similar studies employing *P. gingivalis* to infect rats and to feed them with fish oil in their food (24.6%–35% of ω-3) for 8 weeks demonstrated lower alveolar bone loss [22, 27]. Also, alveolar-bone resorption was reduced in the same model with an extended ω-3 PUFA supplementary diet (22 weeks) [27]. Moreover, the present study assessed the impact of alveolar-bone loss. The results showed that high dose ω-3 supplementation (40 mg/kg (60% EPA, 40% DHA for 71 days) decreases alveolar-bone loss. It has been reported that the effect of ω-3 on bone metabolism were best observed in prolonged and prophylactic administration [18, 19, 29].

It is known that ω-3 supplementation can change body composition because it increases burned calories by brown fat in hibernating animals and newborns [30]. We measured the body weight before and after ω-3 supplementation, but we did not find changes in weight between groups (Supplementary Fig. 1).

In general, the literature suggests that the anti-inflammatory action of ω-3 PUFA requires a minimal administration period of 14 days, and that the prophylactic supplementation for the same period increases EPA and DHA levels in the cell membrane. Consequently, the cell membrane becomes robust and resistant to lysis [27, 31, 32].

The histological examination corroborated the clinical results. H&E staining was utilized to show the infiltration of inflammatory cells, the loss of connective tissue attachment, and alveolar bone resorption in gingival tissues between the distal root of the first molar and the mesial root of the second molar. Infiltrated inflammatory cells revealed a predominant population of polymorphonuclear neutrophils. Histological evaluation of our model demonstrated that the P + O3 and P groups showed severe and mild inflammation. As observed with Sirius Red staining, ω-3 PUFA supplementation prevented the degradation of collagen fibers; the P + O3 group demonstrated a more significant amount of highly organized collagen fibrils than the P group. Furthermore, destruction of the alveolar bone and the extent of inflammation was prevented with a ω-3-PUFA-supplemented diet. Similar, anti-inflammatory effects of ω-3 PUFA have been reported by Hasturk et al. [33], who applied Resolvin E1 (RvE1), a metabolite of ω-3 PUFA, in a topical form afterward in the region of the *P. gingivalis*-infected ligature; those authors observed that the populations of neutrophils were reduced, as was the tissue damage.

Matrix metalloproteinases, or MMPs, comprise an essential family of metalloendopeptidases that collectively degrade all extracellular matrix components (EMC), including the bone mineral matrix [34, 35]. The family member is divided, in which MPP-2 and -9 are classified as gelatinases with similar biological reactions, and these are generated by macrophages, neutrophils, and osteoclasts [35, 36]. The present study confirmed the positive expression of MMP-2 and -9 in polymorphonuclear cells, giant cells with a foamy cytoplasm, and large multinucleated cells with a foamy cytoplasm near the areas of bone destruction, consistent with the morphology of neutrophils, macrophages, and osteoclasts, in periodontal inflamed tissues. In addition, immunohistochemical expression could be found in the fibroblasts, endothelial cells, keratinocytes, and bone cells of the inflamed tissues in our periodontitis model. In general, MMP transcripts are expressed at low levels, but they rapidly increase when tissue undergoes remodeling in processes such as inflammation, wound healing, and cancer [37].

It is recognized that MMP-2 and -9 can be stored in inflammatory-cell granules but are more often secreted and found anchored to the cell surface or bound to other proteins on the cell surface or within the EMC [37]. The former is consistent with the pattern of expression found in periodontitis-induced tissues; in this regard, DAB staining was observed in areas of collagen fibers under the ligature region. Moreover, the analysis of the expression levels of MMP-2 and -9 exhibited a close relation with infected, inflamed tissues and tissue damage.

The inhibition of MMP activity through ω-3 PUFA in different pathologies has been reported. In mice fed with a high ω-3 PUFA diet, it was demonstrated that MMP-9 immunoreactivity decreased in aortic tissue [38] and, in patients with sclerosis multiple, omega-3 fatty acid supplementation for 3 months decreased MMP-9 levels from peripheral blood mononuclear cells [39]. To the best of our knowledge, this is the first report focusing on the inhibitory effect of ω-3 PUFA supplementation and immunohistochemical expression of MMP-2 and -9.

Advances in immunology unravel the fact that the loss of periodontal supporting tissues is a consequence of the host’s immune response and that the role of cytokines is critical [40, 41]. The majority of the cytokines investigated regarding periodontitis pathogenesis are pro-inflammatory [40]. For example, IL-1β has been studied extensively, followed by TNF-α and IL-6. By contrast, few papers have evaluated anti-inflammatory cytokines, with the majority of research being conducted on IL-4 and IL-10 [40, 41].

Different results concerning the action of ω-3 PUFA in the decrease of alveolar-bone resorption have been presented. However, the majority of these studies showed an effective anti-inflammatory action in animal models of periodontitis treated with ω-3 PUFA at different doses and in different periods [8]. In animal models, fatty-acid deficiency caused severe osteoporosis, while high fatty-acid intake is associated with lower bone resorption, supporting the assumption that diets high in ω-3 fatty acids are typically understood to support bone health [42, 43].

Nevertheless, the effects of ω-3 PUFA on bone metabolism are not entirely understood. In this regard, different studies suggest that the effects on bone metabolism are better observed in extended ω-3 PUFA administration, which can be corroborated with the results presented herein. It has been suggested that ω-3 PUFA can inhibit the differentiation, activation, and function of osteoclasts, reducing the levels of RANKL induced by pro-inflammatory cytokines, leading to the suppression of inflammatory cytokines and to the activation of NF-κB, a process that results in less bone resorption [19, 27]. In the present study, inflammatory cytokine expression decreased in the serum, and tissue samples demonstrated that MMP-9 staining was visible in the cytoplasm of osteoclast-like cells in the P group. However, expression was decreased in the P + O3 group, which could also be related to osteoclast function.

An essential aspect of the present study was to evaluate the effects of ω-3 PUFA on immune-system regulation, measuring the levels of the pro-inflammatory cytokines TNF-α, IL-2, and IFN-γ, and anti-inflammatory IL-4, and IL-5. In addition to immune-system regulation, the efficacy of prophylactic and therapeutic effects of ω-3 PUFA were evaluated. As in the study performed by Araghizadeh et al. [10], prophylactic supplementation with ω-3 PUFA decreased the expression of TNF-α in mice with ligature-induced periodontitis. In addition, histological analysis showed that supplementation with ω-3 PUFA decreases tissue inflammation. The mechanisms of the anti-inflammatory effects of ω-3 PUFA remain an open question; however, it has been proposed that the cyclooxygenase and lipoxygenase pathways of leukotrienes and prostaglandins, inflammatory mediators derived from the arachidonic-acid cascade, are inhibited by ω-3 PUFA, consequently reducing the levels of pro-inflammatory cytokines such as IL-2, TNFα, and INFγ [8]. Notably, we observed an increase in IL-5 in the P + ω-3 group compared to control mice, which is interesting because ω-3 supplementation in mice with LPS-stimulated systemic inflammation has been shown to increase circulating levels of IL-5, which can exert immunomodulatory effects but could be harmful for inflammatory lung diseases [44]. Therefore, it would be interesting to evaluate the immune mechanisms induced by ω-3 supplementation in local chronic inflammatory diseases such as periodontitis and its long-term impact on Th2 inflammatory diseases. The small amount of information in the literature concerning the use of ω-3 PUFA and the variation in related protocols make it difficult to compare the existing data. Further investigation is necessary to elucidate the mechanism of anti-inflammatory effects of ω-3 PUFA in the oral cavity.

The results of the present study suggest that prophylactic and extended supplementation with ω-3 PUFA may be used as a preventive strategy for periodontitis or alveolar bone loss in patients at risk of developing periodontal disease. Based on the systemic anti-inflammatory effects of ω-3 PUFA supplementation, it could be assumed that there is a positive host modulation of inflammatory bone destruction and of the MMP-2 and MMP-9 activity caused by periodontitis. It could be important to study the incidence or severity of periodontitis in individuals with western diets (rich in ω-3) who could benefit from this.

## Conclusion

The results presented herein demonstrated that long-term supplementation with ω-3 PUFA alone is sufficient to protect bone resorption in our murine model, probably by decreasing MMP-2 and -9 and its immunoregulatory properties.

## Data Availability

The datasets used and/or analysed during the current study are available from the corresponding author on reasonable request.
